# Leveraging NKG2D Ligands in Immuno-Oncology

**DOI:** 10.3389/fimmu.2021.713158

**Published:** 2021-07-29

**Authors:** Mercedes Beatriz Fuertes, Carolina Inés Domaica, Norberto Walter Zwirner

**Affiliations:** ^1^Laboratorio de Fisiopatología de la Inmunidad Innata, Instituto de Biología y Medicina Experimental (IBYME-CONICET), Buenos Aires, Argentina; ^2^Facultad de Ciencias Exactas y Naturales, Departamento de Química Biológica, Universidad de Buenos Aires, Buenos Aires, Argentina

**Keywords:** NK cells, NKG2D, MICA, tumor immunity, immuno-oncology

## Abstract

Immune checkpoint inhibitors (ICI) revolutionized the field of immuno-oncology and opened new avenues towards the development of novel assets to achieve durable immune control of cancer. Yet, the presence of tumor immune evasion mechanisms represents a challenge for the development of efficient treatment options. Therefore, combination therapies are taking the center of the stage in immuno-oncology. Such combination therapies should boost anti-tumor immune responses and/or target tumor immune escape mechanisms, especially those created by major players in the tumor microenvironment (TME) such as tumor-associated macrophages (TAM). Natural killer (NK) cells were recently positioned at the forefront of many immunotherapy strategies, and several new approaches are being designed to fully exploit NK cell antitumor potential. One of the most relevant NK cell-activating receptors is NKG2D, a receptor that recognizes 8 different NKG2D ligands (NKG2DL), including MICA and MICB. MICA and MICB are poorly expressed on normal cells but become upregulated on the surface of damaged, transformed or infected cells as a result of post-transcriptional or post-translational mechanisms and intracellular pathways. Their engagement of NKG2D triggers NK cell effector functions. Also, MICA/B are polymorphic and such polymorphism affects functional responses through regulation of their cell-surface expression, intracellular trafficking, shedding of soluble immunosuppressive isoforms, or the affinity of NKG2D interaction. Although immunotherapeutic approaches that target the NKG2D-NKG2DL axis are under investigation, several tumor immune escape mechanisms account for reduced cell surface expression of NKG2DL and contribute to tumor immune escape. Also, NKG2DL polymorphism determines functional NKG2D-dependent responses, thus representing an additional challenge for leveraging NKG2DL in immuno-oncology. In this review, we discuss strategies to boost MICA/B expression and/or inhibit their shedding and propose that combination strategies that target MICA/B with antibodies and strategies aimed at promoting their upregulation on tumor cells or at reprograming TAM into pro-inflammatory macrophages and remodeling of the TME, emerge as frontrunners in immuno-oncology because they may unleash the antitumor effector functions of NK cells and cytotoxic CD8 T cells (CTL). Pursuing several of these pipelines might lead to innovative modalities of immunotherapy for the treatment of a wide range of cancer patients.

## Introduction

The therapeutic alternatives to treat tumors received a formidable boost when immune checkpoint inhibitors (ICI) were developed (1). These assets revolutionized the field of immuno-oncology (I-O), leading to successful treatment of liquid and solid tumors. ICI are monoclonal antibodies (mAb) whose mechanism of action involve the blockade or interference with cell surface receptors that mediate inhibitory signals in cells of the immune system, mainly T lymphocytes but also natural killer (NK) cells (2, 3). However, ICI also have a dark side that comprises a low frequency of responding patients, their adverse effects (4) and, more recently described and still ill-characterized, the occurrence of hyper-progression of the tumor (5–7). An additional promising option is to combine ICI with other anti-tumor compounds. Combination of anti-CTLA4 and anti-PD-1 or anti-PD-L1 mAb has shown promising results in several tumors (8). Moreover, novel strategies, molecular targets and cell-based therapies keep emerging as alternatives to improve the efficacy of the treatment options for cancer patients, positioning the field of I-O at the top in the investment and competition for academic institutions and pharmaceutical/biotech companies.

## NK Cells at the Forefront in Immuno-Oncology

NK cells and CTL constitute the most relevant effector cells that mediate tumor cell elimination through their cytolytic activity and a proinflammatory function. Cytolytic activity is exerted though the secretory pathway and the expression of death receptors (9). Inflammatory activity is exerted through the secretion of cytokines, mainly IFN-γ and TNF, and chemokines such as CCL5, XCL1 and XCL2 (10, 11). Evidence obtained some years ago (12), and recent studies that established that NK cell frequency, infiltration and function are associated with improved patient survival (13–16) demonstrated that NK cells play a crucial role in tumor immunity. Remarkably, although NK cells were considered rapid but short-lived responders to virus-infected and tumor cells, this idea has been challenged by the identification of NK cells that undergo clonal expansion and acquire features of long- lived memory cells, a hallmark of adaptive T and B lymphocytes. The molecular and cellular mechanisms that drive adaptive NK-cell expansion and activity are being elucidated (17). These concepts strengthened even more the interest in exploiting NK cells´ immunotherapeutic potential to combat cancer in oncologic patients (18, 19).

Furthermore, NK cells became progressively positioned at the forefront of current immunotherapy strategies (18, 19). Many new compounds, including but not limited to mAb are being developed to fully exploit their antitumor potential (20). Also, it is currently possible to produce large amounts of NK cells suitable for adoptive transfer to patients. A recapitulation of NK cell-based therapies in I-O indicates that most of these approaches fall within one of the following categories: *a) in vitro* expansion and activation; *b)* adoptive transfer of allogeneic NK cells; *c)* generation of chimeric antigen receptor modified NK cells (CAR-NK) and *d)* administration of mAb or other bioactive compounds that regulate NK cell activity against tumors (21). Some success in the treatment of liquid tumors has been achieved using these NK cell-based strategies (22–27). While *in vitro* expansion and activation of autologous NK cells, and adoptive transfer of allogeneic NK cells have yielded variable degrees of success with liquid tumors, high hopes have been put on the generation and use of CAR-NK. This is because CAR-NK cells have several advantages over CAR-T cells such as a shorter half-life (and a subsequent better opportunity to control eventual side effects), a lack of induction of cytokine release syndrome (CRS, often severe and/or fatal in patients that received CAR-T cells), and the possibility of preparing off-the-shelf CAR-NK cells for the treatment of multiple patients (28–30). However, the landscape is quite different for solid tumors mostly because NK cells must face the formidable task of overcoming the immunosuppressive TME to avoid becoming exhausted and dysfunctional (31, 32). Also, even if NK cell can overcome this hostile environment, their weak capacity to infiltrate solid tumors is another of the reasons that explain the low success of NK cell-based therapies to treat solid tumors (28, 29). Thus, adoptive transfer of NK cells might require the combination with additional strategies to bolster an effective anti-tumor NK cell function. Combination with ICI emerge as attractive possibilities but, in view of our current knowledge about dysfunctional NK cells, other molecules such as TIM-3, TIGIT and LAG-3 are taking the center of the stage in I-O, as their blockade, knock down or knock out results in a better tumor eradication in different models (33).

The possibility of promoting NK cell effector functions through immunotherapeutic manipulation is further supported by data that indicate that NK cells respond to ICI. Single-cell RNA sequencing (scRNAseq) data indicate that tumor NK cell infiltration is associated with better patient outcomes in several different cancer types (13, 15) and that NK cell infiltration contributes to a robust ICI response (10, 14). Also, scRNAseq and CYTOF revealed that ICI induced a significant remodeling of lymphoid and myeloid cells in the TME, and this effect was dependent on IFN-γ (34). Accordingly, there is a considerable interest in harnessing antitumor NK cell effector functions through the development of novel cancer immunotherapies (21, 35). Many companies currently have NK cell pipelines in their portfolios mainly intended to foster NK cell effector functions in cancer patients using novel ICI or immunomodulatory agents (35–37). However, these strategies face the challenge of having to overcome the decline in NK cell activity due to tumor immune escape mechanisms. In addition, in ccRCC, an RNAseq analysis demonstrated that expression of NK cell-associated receptors and molecules, and some other ligands recognized by these receptors affect overall survival (38). These findings sustain the necessity of a deeper exploration of the TME as a major contributor to NK cell (dys)function and the characterization of tumor-specific factors and mechanisms that regulate NK cell activity. Additionally, a big question is whether it is feasible to reinvigorate dysfunctional tumor-infiltrating NK cells (TINK) or to eliminate/deplete them and create a niche for the recruitment of newly activated, fully functional NK cells through the administration of immunotherapeutic agents to the patient.

## The NKG2D Receptor and Its Ligands

NK cells detect tumor cells through a collection of germline-encoded activating receptors whose actions are counterbalanced by another group of inhibitory receptors (28, 39, 40). Thus, NK cells develop effector functions when signals triggered by activating receptors override signals triggered by inhibitory receptors. NK cell activity is also regulated by cytokines, especially those produced by myeloid cells such as IL-12, IL-23, IL-27, IL-15, IL-18 and TGF-β (41–44) and by agonists of several Toll-like receptors (TLR) such as TLR3, TLR7 and TLR9 that are expressed by NK cells (45–47). Therefore, the integration of activating and inhibitory signals present in their environment dramatically determines NK cells’ capacity to mobilize effector functions.

Besides CD16 (FcγIIIa receptor) that recognizes Fc fractions of several IgG subclasses and is responsible for the antibody-dependent cell-mediated cytotoxicity (ADCC) (48), the Natural Cytotoxicity Receptors (NCR) NKp30 (CD337, the product of the *ncr3* gene), NKp44 (CD336, the product of the *ncr2* gene), NKp46 (CD335, the product of the *ncr1* gene) and NKp80 (the product of the *klrf1* gene), together with DNAM-1 (CD226) and NKG2D (CD314, the product of the *klrk1* gene) emerged as a major activating receptors (17, 37, 49–52). *In vivo* blockade of NKG2D or NKG2D knock out mice leads to an increased susceptibility to spontaneous tumor development and tumor progression (53, 54). Therefore, efforts are underway to capitalize on NKG2D ligands (NKG2DL) as molecular targets in I-O. In humans, 8 different NKG2DL have been described (51, 55). The first known NKG2DL were the proteins encoded by the *MHC class I-chain related genes A* and *B* (*MICA* and *MICB*), also called *PERB11.1* and *PERB11.2* respectively (56, 57). Both genes map within the MHC, are highly polymorphic (58, 59) and the alleles are expressed in a codominant manner (60). The MICA and MICB proteins encoded by most alleles consist of three extracellular domains, one transmembrane domain and a cytoplasmic tail. An exception is the MICA*008 allele (the most frequent in different populations) that harbors an insertion in exon 5 that introduces a shift in the reading frame, encoding a truncated protein with a partial transmembrane domain and no cytoplasmic tail. Nonetheless, MICA*008 is stably expressed on the cell surface of different cells. MICA and MICB are also highly glycosylated. Due to their polymorphic nature, MICA and MICB constitute targets of the immune response against allogeneic transplants and patients with kidney, hearth and lung transplant rejection exhibit anti-MICA/B Ab in serum (61–66).

Although the significance of the polymorphism of MICA and MICB remains ill-defined, associations between alleles and autoimmune diseases and cancer has been widely reported (67–75). Also, a differential regulation of cell surface expression of MICA isoforms has been observed upon infection with cytomegalovirus (76), suggesting that resistance to infectious agents could be a driving force for the selection of several MICA alleles in the population. Dimorphism at position 129, which maps to the α2 domain of MICA, affects NKG2D recognition. Alleles with Met at position 129 trigger stronger NKG2D signaling and subsequent NKG2D-dependent effector functions than alleles with Val, but, at the same time, MICA-129Met isoforms promote a more intense downregulation of NKG2D than MICA-129Val isoforms (77). MICA-129Met homozygosity confers susceptibility to inflammatory bowel disease (78), while MICA-129Val homozygosity leads to faster progression of multiple myeloma (MM) (79) and also plays a role in susceptibility for breast cancer development (80). Therefore, although the majority of the polymorphic positions in MICA are not exposed to its contact area with NKG2D (58, 81), such polymorphism may affect other aspects of MICA and determine the chances of success of strategies aimed at leveraging MICA as molecular target in I-O.

The existence of MICA-null haplotypes in individuals without particular susceptibility to infectious or autoimmune diseases or cancer indicates some redundancy in the biological function of MICA (82–85). Likely, this is because there are additional ligands for NKG2D. Besides MICB, which shares a homology of above 80% with MICA, currently we know six additional NKG2DL but with a homology with MICA and MICB that is below 25%. These additional NKG2DL are members of the UL-16 binding protein (ULBP) family, also known as Retinoic Acid Early Transcripts (RAET) 1. Therefore, they were named ULBP-1 (or RAET1I), ULBP-2 (or RAET1H), ULBP-3 (or RAET1N), ULBP-4 (or RAET1E), ULBP-5 (or RAET1G) and ULBP-6 (or RAET1L) (51, 55, 86–89).

It has been considered for some time that MICA and MICB are not expressed or are weakly expressed in normal cells with very few exceptions such as the luminal side of the intestinal epithelium (56, 90–93). However, it was observed later that MICA and MICB transcripts could be detected in most normal tissues with the exception of the central nervous system (94). However, as NKG2DL experience post-translational modifications that regulate their expression as cell surface molecules (95–97), their cell surface expression on normal cells remains controversial. We demonstrated that premalignant quiescent melanocytic nevi, benign lesions of the skin and normal skin do not express MICA, in opposition to a primary recently diagnosed melanoma (98). Also, expression of MICA/B in many tumors and normal epithelia was recently reported, but with a predominant intracellular localization and low cell surface expression (99). The reasons for these discrepancies are not quite well understood but different technical approaches to assess cell surface MICA expression may produce discrepant results. As MICA colocalizes with some intracellular markers, these results indicate that there is an intracellular pool of MICA. Moreover, cell surface expression should be better analyzed by flow cytometry instead of tissue microscopy to generate compelling information. Accordingly, we observed that several melanoma cell lines and metastatic melanomas display an intracellular pool of MICA but only some of them exhibit cell surface MICA (100).

**Figure 1:**
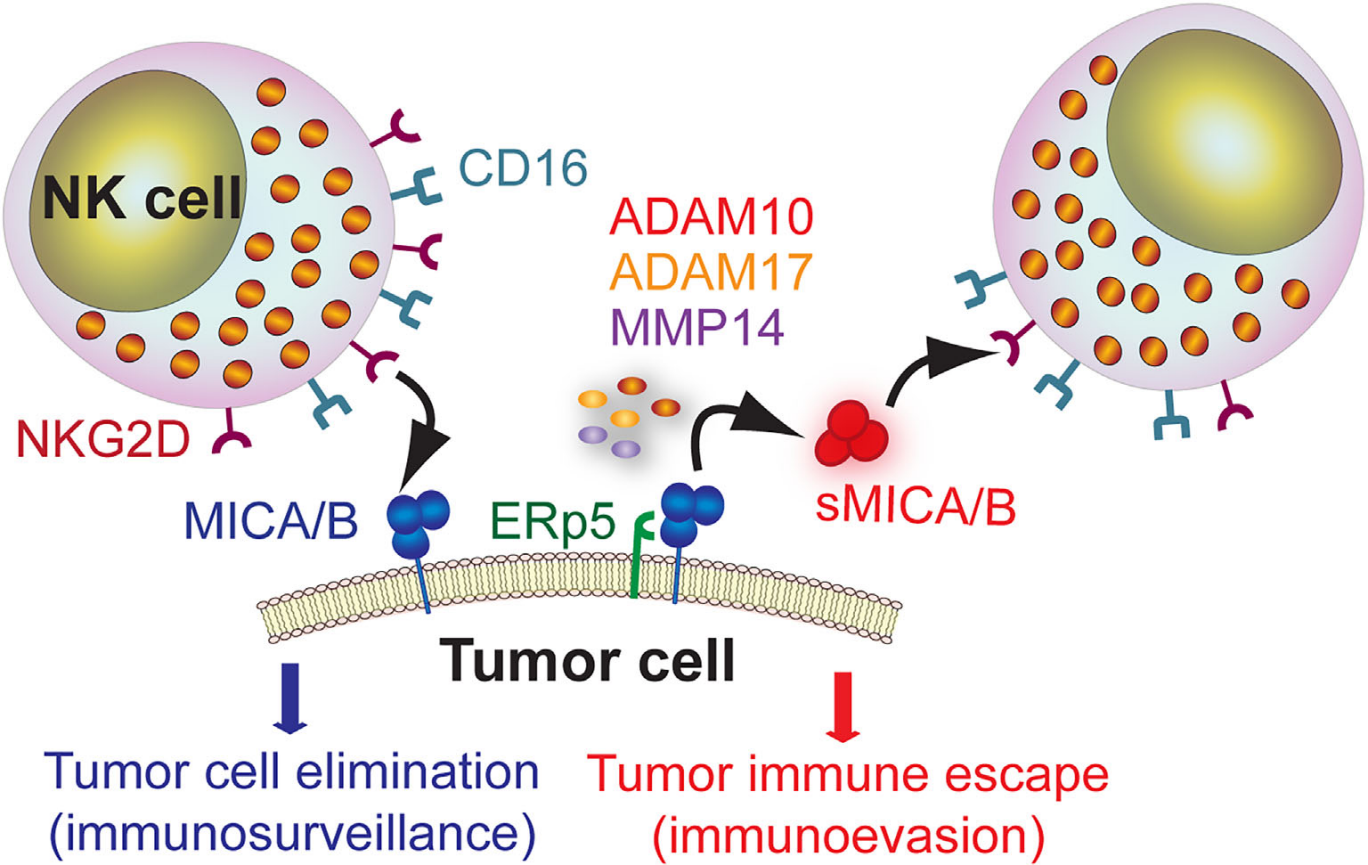
Dual role of MICA/B as target molecule for immunosurveillance by NK cells and as mediator of tumor immune escape. MICA/B expressed on the cell surface of tumor cells can be recognized by NK cells through NKG2D and promote a cytotoxic response that leads to tumor cell elimination (immunosurveillance). However, MICA/B can associate with the ERp5 chaperone and, through a proteolytic cleavage mediated by ADAM10, ADAM17 and MMP14, generate sMICA/B that promote NKG2D down-regulation and impairment of NK cell-effector functions, thus facilitating tumor immune escape (immunoevasion).

MICA is expressed in a wide variety of tumors (91, 101–110). RNA sequencing (seq) data indicate that MICA is the NKG2DL that exhibits the highest expression in, from example, lung, colorectal, stomach, liver and breast cancers (111). Also, MICA exhibits a very low tumor mutational burden, suggesting that its expression is not subject to DNA editing to confer some kind of adaptive advantage to tumors. Although over-expression of NKG2DL may represent a valid strategy to limit tumor progression (112–114), tumors display escape strategies that subvert the biological function of NKG2D (115, 116). The underlying mechanisms involves the proteolytic shedding of MICA and other NKG2DL induced by tumor-secreted metalloproteases (MMP) (115–117) or secretion in exosomes (118). Released soluble MICA (sMICA) and soluble MICB (sMICB) can thereafter bind to NKG2D and induce its down-modulation and degradation, subverting NKG2D-dependent effector functions of NK cells and facilitating tumor immune escape (110, 115, 116). This dual role of MICA/B is schematically represented in **Figure 1**. However, recent data indicates that the suppressive activity of sMICA on NK cells is not due to the down-regulation of NKG2D but to a blockade of NKG2D by sMICA (119). In addition, other mechanisms account for low cell surface expression of MICA and impaired recognition by NKG2D, as we have demonstrated previously (100).

The different UBLP or RAET1 also have been shown to be over-expressed on tumors (mainly on leukemic blasts) and mobilize NKG2D-dependent NK cell effector functions (110, 120–126). These additional NKG2DL, unlike MICA and MICB, consist of two extracellular domains. ULBP-4 and ULBP-5 also carry a transmembrane domain and a cytoplasmic tail. However, ULBP-1, ULBP-2, ULBP-3 and ULBP-6 are anchored to glycosylphosphatidylinositol (GPI) of the cell membrane (127) and ULBP-5 can also generate a GPI-anchored form (128). Also, ULBP can generate soluble forms with immunosuppressive activity due to a shedding process mediated by MMP, by phosphatidylinositol phospholipase C or in exosomes (129–132).

Moreover, NKG2D plays an important role during immunosurveillance in patients with acute myelogenous leukemia (AML) (133). AML mortality is mostly due to recurrence caused by chemotherapy-resistant leukemia stem cells (LSC), which, in contrast to bulk AML cells, are highly leukemogenic in immunodeficient mice (134). NKG2DL are broadly expressed on patient-derived bulk AML cells but not on LSC. However, when LSC differentiate into mature AML cells, they upregulate NKG2DL expression, and these NKG2DL-expressing AML cells, in opposition to LSC, become susceptible to NK cells *in vitro*. Therefore, LSC may preclude NK cell effector functions through suppression of NKG2DL expression and contribute to the AML burden through a continuous differentiation into NKG2DL^+^ AML blasts. Hence, therapy-induced upregulation of NKG2DL in LSC from AML may promote the reinstatement of susceptibility to NK cells.

The reason for the multiplicity of NKG2DL remains speculative. Although all of them seem to be over-expressed by different tumor cells, more knowledge has been obtained for MICA and MICB. These two NKG2DL also stand out because potential therapeutic strategies targeting MICA and MICB but not therapeutic strategies targeting UBLP emerged during the last years. Therefore, we focused this review mainly on MICA and MICB as targets in I-O.

## The Problem of the TME and TAM as Tumor Protective Shield

Given the formidable challenge of overcoming the immunosuppressive TME to leverage NK cell-based immunotherapies for solid tumors (28, 29), it becomes necessary to elucidate the regulatory circuits that shut down NK cell effector functions in this particular niche. NK cells can function as drivers of tumor inflammation, which leads to tumor immune cell infiltration and promotes the conversion of “cold” tumors into “hot” tumors, subsequently conferring better responsiveness to ICI (10). NK cells´ critical functions to induce an effective tumor immunity depend on a successful crosstalk with conventional dendritic cells (cDC1) and the production of the chemokines CCL5 and XCL1, and that tumor-derived prostaglandin E2 (PGE2) interferes with this reciprocal stimulatory loop (11). In addition, early accumulation of IFN-γ-producing TINK promote the remodeling of the TME and unleash CTL-mediated tumor eradication, a circuit that is interfered by tumor-derived prostaglandin E2 (PGE2) acting *via* EP2 and EP4 receptors on NK cells (135). Moreover, only tumors responsive to ICI exhibited an inflammatory gene expression signature after ICI treatment accompanied by increased infiltration by NK cells that displayed an activated phenotype and produced IFN-γ, and that were critical for this response (136).

Moreover, additional mechanisms and mediators that affect NK cell recruitment, activation and display of optimal effector functions against solid tumors in the TME are known (137–141). TGF-β is a major negative regulator of NK cell effector function and tumor elimination ability (142–148). In LNT-229 glioma cells, it has been observed that an autocrine circuit that involves TGF-β and MMP production promotes shedding of MICA and ULBP-2 and negatively affects the expression of these NKG2DL on the tumor cell surface (149). TGF-β also negatively affects expression of NKp30 and NKG2D on human NK cells stimulated with IL-2 and NK cell-mediated cytotoxicity (150). TGF-β also impacts on NK cell metabolism, inducing a reduced glycolysis and oxidative phosphorylation that dampens NK cell effector functions (151). These effects can be mediated by different forms (soluble or membrane-bound) of TGF-β produced by regulatory T cells (152, 153), anti-inflammatory macrophages (44, 154) or by myeloid-derived suppressor cells (MDSC) (155). Consequently, it has been suggested that novel therapies that interfere with TGF-β may trigger NKG2D-dependent NK cell-mediated tumor elimination (143). Among them, bintrafusp alfa (M7824, a bifunctional fusion protein targeting TGF-beta and PD-L1) and galunisertib have shown promising results. Bintrafusp alfa has been demonstrated to revert TGF-β-mediated suppressive effects on NK cells and is currently explored in different clinical trials (156). Galunisertib, a SMAD2 inhibitor, has been shown to facilitate NK cell activation and effector function against neuroblastomas, and enhance ADCC by dinutuximab (157). Also, it has been observed recently that glioblastomas are infiltrated by NK cells that display an altered phenotype and impaired effector functions. Moreover, treatment of mice engrafted with glioblastoma stem cells (GSC) with allogeneic NK cells and galunisertib or Ly2019761 prevented the development of such dysfunctional NK cells and allowed a better control of tumor growth, indicating that TGF-β was responsible for this effect (158). These data highlight that the TME strongly affects NK cell effector functions and that they can be reinvigorated by therapeutic intervention. MDSC also produce indoleamine 2,3-dioxygenase (IDO), and enzyme that catabolizes tryptophan and participates in a metabolic pathway that generates kynurenine, and this pathway impairs NK cell effector functions (159, 160). Another soluble mediator that blunts NK cell effector function is PGE2, which is abundant in the TME of several tumors (11, 161–168).

Another factor that contributes to NK cell dysfunction is chronic stimulation. Mice with ubiquitous enforced expression of MICA that triggered chronic stimulation through NKG2D exhibited NK cells with reduced expression of NKG2D, and these NK cells exhibited an impaired NKG2D-dependent NK cell-mediated cytotoxicity and accelerated growth of a MICA-expressing melanoma (169, 170). In humans, it has been observed that chronic HCMV infection leads to an increased frequency of CD57^+^NKG2C^+^ peripheral blood NK cells (PBNK) that display rapid and robust effector function upon restimulation. However, their chronic stimulation induced high expression of the co-inhibitory receptors LAG-3 and PD-1, and these chronically stimulated NK cells were dysfunctional due to epigenetic reprograming and alterations in DNA methylation (171). Therefore, chronic stimulation of NK cells through several activating receptors, including NKG2D, leads to a functional impairment that can impact negatively on NK cell-mediated immunosurveillance.

TAM constitute major components of the TME and players of tumor immune escape. Strategies aimed at targeting TAM to promote their elimination or reprogramming may lead to better clinical outcome especially in patients with solid tumors (172). Interference with TAM-mediated immunosuppression targeting the scavenger receptor MARCO with a blocking mAb lead to decreased tumor vascularization, metabolic reprogramming of TAM, and an efficient activation of NK cell cytotoxic effector functions, and this effect also synergized with ICI (173). Therefore, targeting TAM can improve tumor immunity through restoration of NK cell activation and effector functions, further supporting the idea that strategies that convert “cold” tumors into “hot” tumors through manipulation of TAM and NK cells might constitute forefront strategies in I-O.

## NK Cell Reinvigoration Through Targeting Co-Inhibitory Receptors

The possibility to phenotypically characterize dysfunctional TINK generated a renewed interest in targeting co-inhibitory receptors to reinvigorate NK cells in cancer patients using current or novel ICI (174). Targeting the PD-1/PD-L1 axis, CD96 (TACTILE), NKG2A, TIGIT, TIM-3 and LAG-3 have emerged as forefront alternatives because they are usually over-expressed in dysfunctional NK cells (33, 52, 175–179). Although the revolution in I-O achieved with ICI was originally attributed to an enhanced T cell-mediated antitumor response, increasing evidence demonstrates that NK cells also express PD-1 and PD-L1, and that they constitute targets of ICI that results in a reinvigoration of anti-tumor NK cell effector functions. ICI induced a CTL- and NK cell-mediated tumor growth control accompanied by a weakened suppressive immune cell infiltrate in the TME in a murine model of glioblastoma (180). Moreover, increased frequencies of PD-1^+^ NK cells with heightened expression of PD-1 were observed in PBNK and TINK from patients with several gastrointestinal tumors, and higher expression correlated with impaired survival in some cases. These NK cells exhibited impaired IFN-γ production and degranulation, after *in vitro* exposure to ICI led to a functional reinvigoration, and *in vivo* treatment with ICI of nude mice xenografted with a human esophageal squamous cell carcinoma caused NK cell-dependent delayed tumor growth (181). TINK from transplantable, spontaneous, and genetically induced tumors also contained a high frequency of PD-1^+^ NK cells and suppressed IFN-γ production and cytotoxicity *in vitro* but ICI treatment resulted in a restoration of NK cell response that was essential for the therapeutic effect ICI (182). ICI also exert effects beyond their blocking activity as an anti-PD-L1 mAb such as avelumab, but no atezolizumab, can trigger ADCC by PBNK from healthy donors against triple negative breast cancer (TNBC) tumor cell lines *in vitro* (183). Also, ADCC triggered by anti-PD-L1 mAb against multiple carcinoma cell lines could be enhanced by histone deacetylase inhibitors (HDACi) because they augmented the expression of PD-L1, NKG2DL and other NK cell activating ligands and death receptors on target cells (184). Chemotherapeutic agents can increase the susceptibility of nasopharyngeal carcinoma cell lines to NK cell-mediated cytotoxicity, and such effect was enhanced by an anti-PD-1 blocking mAb because the chemotherapeutic agents also stimulated up-regulation of PD-1 on NK cells and PD-L1 on the target cell lines (185). In head and neck cancer patients, PBNK contain a higher frequency of PD-1^+^ cells, and ICI treatment with an anti-PD-1 mAb promotes heightened cetuximab-mediated NK cell activation (186). In non−small cell lung cancer patients, TINK exhibit functional defects accompanied by a higher frequency of PD-1^+^ cells, while *in vitro* treatment with ICI reverted such NK cell dysfunction (187).

Targeting other co-inhibitory receptors on NK cells is also under investigation. TIGIT is a co-inhibitory receptor that emerged as ICI candidate because it is over-expressed on exhausted TINK and tumor-infiltrating T cells and is responsible for such functional exhaustion. Blockade of TIGIT induced a NK cell reinvigoration, restored an efficient tumor immunity, and also enhanced the efficacy of therapy with ICI against PD-L1 (188). CD96 is another co-inhibitory receptor expressed on NK cells that, through binding to CD155 expressed on tumor cells, limits NK cell effector functions (189). Patients with hepatocellular carcinoma that present reduced disease-free survival have dysfunctional (exhausted) TINK with a higher frequency of CD96^+^ cells and increased expression of CD96, but blockade of CD96 restores NK cell-mediated effector functions (190). NKG2A is another inhibitory receptor that associates with CD94 and negatively regulates NK cell functions. High expression of NKG2A and of its ligand HLA-E can be detected in tumor tissue of hepatocellular carcinoma patients, and NKG2A-expressing TINK exhibit features of exhausted cells and are associated with a poor prognosis (191). NKG2A and HLA-E are overexpressed in several other human cancers including head and neck, colorectal, ovarian, endometrial and cervical cancers. Blocking NKG2A with Monalizumab (a humanized anti-NKG2A mAb) enhances tumor immunity in combination with ICI against PD-L1 by promoting NK cell and CTL cell effector functions in mice and humans and enhances ADCC in combination with Cetuximab against a head and neck carcinoma cell line, indicating that its mechanism of action directly impacts on NK cell effector functions (192–194). Also, patients with chronic lymphocytic leukemia (CLL) exhibit an upregulation of LAG-3 on leukemic blasts, NK cells and T lymphocytes, accompanied by high amounts of soluble LAG-3 (sLAG-3) in plasma, that correlated with impaired outcome. However, *in vitro* exposure of peripheral blood mononuclear cells to relatlimab, an anti-LAG-3 blocking mAb under evaluation in several clinical trials, depleted leukemic cells and restored NK cell- and T cell-effector functions (179). Conversely, the use of a blocking mAb against the inhibitory receptors KIR2DL1, KIR2DL2 and KIR2DL3 named Lirilumab (195, 196) did not show the expected clinical efficacy (197, 198), indicating that target co-inhibitory receptors need to be carefully selected.

Altogether, the described evidences demonstrate that NK cells are dysfunctional in cancer patients and that their reinvigoration appears as feasible, and these findings are motorizing the development of novel ICI aimed at leveraging NK cell mediated effector functions (177). However, targeting co-inhibitory receptors has a dark side as treatment with ICI can trigger many cytokine-mediated immune-related adverse events (irAE) that can be severe and require interruption of ICI treatment and/or complementary treatment with other compounds (199–203). Moreover, the description of patients with accelerated tumor growth after treatment with ICI (hyperprogression) due to ill-defined mechanisms is an additional concern (5–7). These drawbacks are further complicated by the lack of validated predictive biomarkers that would permit the selection of patients that optimally respond to ICI with minimal or no irAE and no hyperprogression (204, 205). Therefore, the development of novel ICI against additional target molecules requires a deep risk mitigation to avoid irAE and hyperprogression.

## The Opportunity for the NKG2D-NKG2DL Axis in Immuno-Oncology

ADCC is one of the major NK cell-mediated effector functions and therapeutic efficacy of several mAb currently used to treat cancer patients depend on their ability to induce ADCC (48, 183, 206–215). Therefore, selection of appropriate molecular targets expressed by tumor cells is a crucial step towards the development of successful immunotherapies to treat cancer patients. As MICA is the NKG2DL more widely overexpressed in tumors, it is reasonable to consider MICA as a frontrunner candidate as target molecule for I-O strategies. A seminal finding about MICA as target in I-O came from the observation that some melanoma patients that received ipilimumab (an anti-CTLA4 mAb) and autologous tumor cells engineered to produce GM-CSF, spontaneously developed anti-MICA Ab that promoted clearing of sMICA from plasma and opsonization of tumor cells for dendritic cell cross-presentation. These effects were associated with a restoration of the expression of NKG2D on NK cells and CTL, a recovery of NKG2D-dependent NK cell effector functions and a better outcome (216, 217). Later, it was observed that MM patients, in contrast to patients with monoclonal gammopathy of undetermined significance (MGUS), also exhibit high titers of anti-MICA Ab that antagonize with the suppressive effects of sMICA and stimulate dendritic cell cross-presentation of malignant plasma cells (108). The serendipitous appearance of anti-MICA Ab with a therapeutic effect prompted us to develop a strategy to actively induce such Ab. To this end, we generated a chimeric protein consisting of the ectodomain of MICA fused to a bacterial immunogenic protein that exhibits adjuvant properties (*Brucella lumazine synthase*, BLS), used this chimeric protein (named BLS-MICA) for the induction of anti-MICA Ab in tumor-bearing hosts, and investigated their therapeutic activity and mechanism of action. BLS-MICA elicited high titers of anti-MICA Ab in mice that *in vitro* recognized MICA naturally expressed on the cell surface of human tumor cells and on MICA-transduced mouse tumor cells. Prophylactic active immunization with BLS-MICA significantly delayed the growth of MICA-expressing tumors in part due to the ability to promote scavenging of sMICA from mouse sera. Passive immunization experiments demonstrated that such effect was mediated by anti-MICA Ab that mediated *in vitro* and *in vivo* ADCC, and that tilted the balance of tumor-infiltrating cells towards an anti-tumoral/pro-inflammatory phenotype characterized by an increased presence of TAM with an M1-skewed phenotype and antigen-experienced CTL (111). Therefore, immunization with BLS-MICA induced therapeutic anti-MICA Ab that constitute a “two-in-one” strategy as they promote tumor elimination by ADCC and interfere with a tumor immune escape through scavenging of sMICA.

Targeting the NKG2D pathway has also been approached using different chimeric proteins where the ectodomain of MICA was fused to single chain Fv (scFv) against other molecules that promoted NK cell-mediated anti-tumor effects (218–220). Although these approaches have shown some preliminary interesting effects, compared to anti-MICA Ab-based strategies, they only promote the bridging between NK cells and tumor cells and do not target, for example, suppressive sMICA, which limits their competitive landscape.

In addition, several anti-MIC mAb also are showing promising preclinical results. A mAb that targets sMICA (B10G5) demonstrated therapeutic effects, alone or combined with an anti-CTLA-4 mAb in mice challenged with MIC transgenic TRAMP (transgenic adenocarcinoma of the mouse prostate) cells. The mechanism of action of this mAb involves ADCC that results in a revitalization of CTL- and Th1-mediated anti-tumor immunity, and remodeling of the TME (221, 222). Other anti-MICA/B mAb that target the α3 domain and inhibit proteolytic shedding were generated. One of these mAb (7C6) inhibited MICA and MICB shedding by human cancer cells, delayed the growth of mouse melanoma and colon carcinoma engineered to express MICA, reduced human melanoma metastases in a humanized mouse model and its mechanism of action involves the stimulation of NKG2D- and CD16-dependent NK cell-mediated effector functions (223). Also, 7C6 acts synergistically with the HDACi panobinostat through stabilizing tumor cell surface MICA/B expression (224) and exhibits synergy with human cytokine-induced NK cells (CIK) *in vitro* (225). A different humanized anti-MICA/B mAb stimulates NK cell-mediated cytotoxicity *in vitro* against primary hepatocellular carcinoma cells (226). In addition, three novel mAb (5E10, 7G10 and 6E1) that recognize the α3 domain of MICA interfere with the immunosuppressive activity of sMICA on human NK cells and stimulate their activation in an Fc-dependent manner due to the formation of immune complexes with sMICA (119). Additionally, the therapeutic efficacy of mAb-mediated neutralization of sMICA (222) or MICA shedding (223) has been shown to negatively affect tumor growth in mouse models.

**Figure 2:**
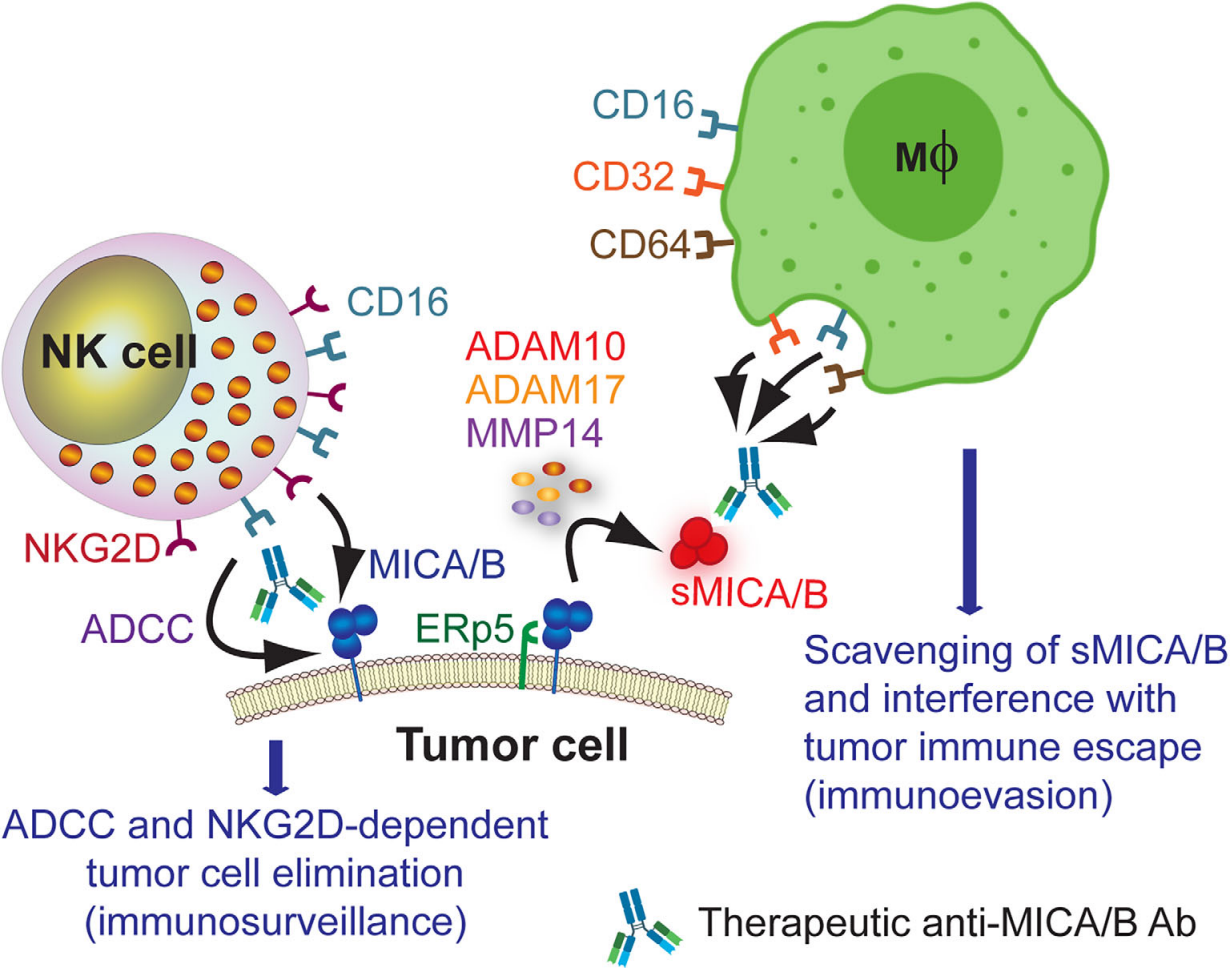
Therapeutic opportunity for anti-MICA/B Ab. Administration of anti-MICA/B Ab may trigger CD16-dependent ADCC by NK cells when these Ab recognize cell surface-expressed MICA/B, contributing to tumor cell elimination. Ab that do not interfere with the binding of NKG2D to MICA/B also would trigger NKG2D-dependent NK cell-mediated cytotoxicity, further contributing to tumor cell elimination. Moreover, recognition of sMICA/B by these therapeutic anti-MICA/B Ab would lead to the formation of immune complexes that would be removed by macrophages upon recognition through CD16, CD32 and CD64. This scavenging of sMICA/B will consequently interfere with tumor immune escape (immunoevasion).

Accordingly, Ab that target MICA/B should trigger ADCC against MICA/B-expressing tumor cells to stimulate tumor cell elimination and, simultaneously, promote the formation of immune complexes with sMICA/B to facilitate their clearance by macrophages and interfere with the tumor immune escape mechanism mediated by sMICA/B. Such Ab will likely display therapeutic activity in patients with tumors that express MICA/B on their cell surface, tumors that shed significant amounts of sMICA or both. This therapeutic opportunity for anti-MICA/B Ab is schematically represented in **Figure 2**. Currently, we cannot anticipate if Ab-based therapies against MICA/B may trigger antigen down-modulation on tumor cells and/or the selection of resistant tumor cells that lost expression of NKG2DL because of a selective killing of tumor cells that express the target molecule. We also cannot anticipate if Ab against NKG2DL promote off target and side/unwanted effects in humans. Also, currently there is not a good estimate about the relative relevance of tumor cell surface-expressed MICA/B *vs* sMICA/B during tumor immunity. The relative expression of MICA/B on the tumor cell surface and the shedding of sMICA/B will probably vary from tumor to tumor and be associated with the ability of each tumor to produce sMICA/B (either by the action of MMP or released in exosomes) and to sustain cell surface MICA/B expression in an immunocompetent host (to resist NK cell effector functions). Therefore, targeting both forms of MICA/B (soluble or cell surface-expressed) with Ab-based strategies emerges as a “two-in-one” strategy that can boost ADCC (immunosurveillance) and that can interfere with the immunosuppressive effect of sMICA/B (tumor immune escape).

Targeting NKG2DL has also been addressed using chimeric antigen receptor (CAR) T cells (CAR-T) or NK cells (CAR-NK). The similarities and differences in using CAR-T or CAR-NK cells have been reviewed recently and one aspect that stands out is that CAR-NK cells appear superior to CAR-T cells to treat hematological malignancies (30). Notably, a big success represents the use of CAR-NK cells directed against CD19, as they induce tumor regression without the development of major toxic effects in the majority of patients with relapsed or refractory CD19-positive leukemias or lymphomas (24). However, treatment of solid tumors with CAR-T or CAR-NK cells has not yielded the expected results, mainly because they must face the immunosuppressive TME (29). Some CAR-T cells engineered to express NKG2D with increased effector functions against cell lines *in vitro* and against xenografted human or syngeneic mouse tumor cell lines *in vivo* have been developed (227–238). Also, CAR-NK cells that express the ectodomain of human NKG2D fused with DAP12 exhibit augmented NK cell-mediated cytotoxicity against several solid tumor cell lines *in vitro* and mediate a therapeutic effect *in vivo* in NSG mice challenged with human tumor cell lines. Moreover, in three human patients with colorectal cancer, these CAR-NK cells mediated tumor regression and patient improvement, highlighting their potential therapeutic utility in solid tumors (239). Although clinical trials for CAR-T and CAR-NK are underway. we don´t know their outcome yet (details can be found at clinicaltrials.gov searching for “CAR-T cells” or “CAR-NK” cells and “NKG2D”). Nevertheless, problems and disadvantages of the CAR-T or the CAR-NK cell approaches are the necessity of promoting the correct migration of the injected cells to the tumors, the long-term persistence of these cells with preserved anti-tumor effector functions, the risk of inducing off target effects, the elevated cost of these alternatives, and the fact that they do not target sMICA/B and consequently, their biological activity may also become vanished in patients with high sMICA/B. In addition, CAR-T cells have the risk of inducing CRS. Therefore, approaches that target the NKG2D-NKG2DL axis, especially MICA/B, with Ab appear superior or with better chance to move to the clinic.

## Rational Design of Combination Therapies That Target the NKG2D-NKG2DL Axis

The current trend towards the use of combination therapies with ICI (240) certainly creates a new landscape to capitalize the therapeutic utility of anti-MICA/B Ab through combination with other agents. Leveraging MICA/B in I-O represents a promising alternative, but these strategies must face the formidable task of having to overcome the suppressive TME including the negative regulatory circuits imposed by TAM. Among recently emerged combination therapies to treat cancer patients, small molecules are taking the center of the stage (241). Several therapies targeting selected molecules and/or mechanisms can be envisaged to be rationally combined with Ab against MICA/B and, consequently, leverage the therapeutic effect of these anti-MICA/B Ab. These strategies would convert “cold” tumors into “hot” tumors through manipulation of NK cell effector functions, the TME (242) and/or TAM (243). In addition, administration of IL-15 leads to huge NK cell expansion in human patients, a fact that might exploit the immunotherapeutic potential of NK cells further (244). Promising candidates that could be combined with anti-MICA/B Ab are described in the following sub-sections.

### Drugs That Can Promote Upregulation of MICA/B on Tumor Cells

NKG2DL expression is controlled by the DNA damage response pathway (DDR) (245), but epigenetic remodelers such as HDACi also triggered upregulated expression of MICA/B (246, 247). Some HDACi such as Trichostatin A (TSA), suberanilohydroxamic acid (SAHA or vorinostat), PXD101 (belinostat), LBH589 (panobinostat) and LAQ824 (dacinostat) are broad spectrum HDACi; others such as valproic acid (VPA), sodium butyrate (NaB), trapoxin and apicidin exhibit specificity for certain groups of HDAC; and others such as MS-275 (entinostat) and FR901228 (romidepsin, depsipeptide or FK228) exhibit high specificity for certain HDAC (248, 249). HDACi exert antiproliferative effects through the induction of cell-cycle arrest, apoptosis, and autophagy, but it has been observed that TSA (247, 250–253), SAHA (247, 254–257), belinostat (247), VPA (246, 251, 252, 254, 258–270), NaB (251, 252, 255, 257, 258, 261, 271), romidepsin (247) and entinostat (255, 257, 269) trigger up-regulation of MICA/B in different cell lines derived from liquid and solid tumors. In most cases, it was demonstrated that HDACi induce upregulation of MICA/B accompanied by a higher NKG2D-dependent, NK cell-mediated cytotoxicity against HDACi-treated tumor cells (246, 247, 250, 251, 253, 255, 257–259, 261–266, 271). In other cases, NKG2D-dependent, CD8 T cell-mediated (260) or γδ T cell-mediated cytotoxicity (270) against HDACi-treated tumor cells was observed. Cooperative effect between HDACi and other compounds such as hydroxyurea (263), gemcitabine (268), All-Trans-Retinoic Acid (ATRA) (260) to promote increased expression of MICA/B was also observed. Moreover, impaired NK cell recognition of vemurafenib-treated BRAF^V600E^ mutant melanoma cells is due to a drug-induced down-regulation of MICA and CD155, while treatment with NaB promotes a recovered surface expression of MICA and NK cell degranulation (272). However, most studies were performed *in vitro*, and only tumor cells were exposed to HDACi. Studies in xenografted immunodeficient mice injected with human tumor cell lines demonstrated that treatment with TSA or VPA and HDACi-mediated upregulation of MICA/B resulted in a delayed tumor growth when they were adoptively treated with cytokine-induced killer cells (251) or with NK-92 cells (266). Moreover, chronic exposure to vorinostat and HDACi-mediated upregulation of MICA/B resulted in a reduced tumorigenic capacity of human colon adenocarcinoma cells xenografted in nude mice (256). Besides, several deleterious effects of HDACi on NK cells were described, such as downregulation of activating receptors and a negative impact on NK cell effector functions (252, 254, 267, 269). Therefore, a delicate selection of HDACi with selective specificity for certain HDAC may lead to an up-regulation of MICA/B without compromising NK cell´s ability to detect tumor cells and mobilize effector functions. Accordingly, VPA down-regulated NKG2D expression and NK cell degranulation but entinostat, which is highly selective for class I HDAC, induced an up-regulated expression of NKG2D and increased NK cell degranulation (269). Moreover, apicidin has been shown to promote the upregulation of ADAM10, one of the MMP involved in MICA/B shedding (273). Therefore, HDACi may induce heightened expression of MICA/B but some of them also increase the amount of sMICA/B. Overall, integrating data about the effect of HDACi on MICA/B expression, on NKG2D expression and on NK cell effector functions, and taking into consideration the few HDACi approved for the treatment of human patients, vorinostsat, belinostat and entinostat emerge as frontrunners to be used to induced heightened expression of MICA/B and sensitize tumor cells to anti-MICA/B Ab.

Upregulation of MICA/B expression can also be achieved with Bortezomib, a proteasome inhibitor that is used to treat patients with MM. MM results from the progression to malignancy of MGUS and MICA expression on malignant plasma cells is higher in MGUS than in MM. However, MM, but not MGUS patients exhibit high sMICA, which is associated with lower expression of NKG2D and NK cell dysfunction in MM patients. Bortezomib promoted upregulated expression of MICA in some MM cells and enhanced the therapeutic effect of anti-MICA Ab that these patients generated spontaneously. Therefore, Bortezomib can cooperate with anti-MICA Ab to exert a therapeutic effect (108). Increased expression of MICA upon *in vitro* or *ex vivo* exposure of MM cells to Bortezomib, accompanied by a subsequent stimulation of NK cell effector functions was also observed by other authors (274, 275). The effect of Bortezomib on MICA expression was also detected on melanoma cell lines (100) and B cell acute lymphocytic leukemias (265), while this drug also promoted the upregulation of MICB on human lung cancer, hepatoma and melanoma cell lines, resulting in an improved NKG2D-dependent NK cell-mediated cytotoxicity (276, 277).

Other drugs used to treat patients with cancer such as sorafenib and sunitinib (278) and 5-fluorouracil (279) affect MICA/B expression. Also, doxorubicin and melphalan, two drugs used to treat patients with MM, induced the expression of MICA/B on cell lines and patient-derived plasmablasts through activation of the DDR pathway, and this effect stimulated heightened NK cell degranulation (274). Another drug that induced MICA/B expression is temozolomide (TMZ), a drug used in some patients with glioblastomas. TMZ upregulated the expression of MICA/B *in vitro* and *in vivo* in murine and human glioblastoma models, and this effect facilitated a NGK2D-dependent elimination of the glioblastoma cells (280).

**Figure 3:**
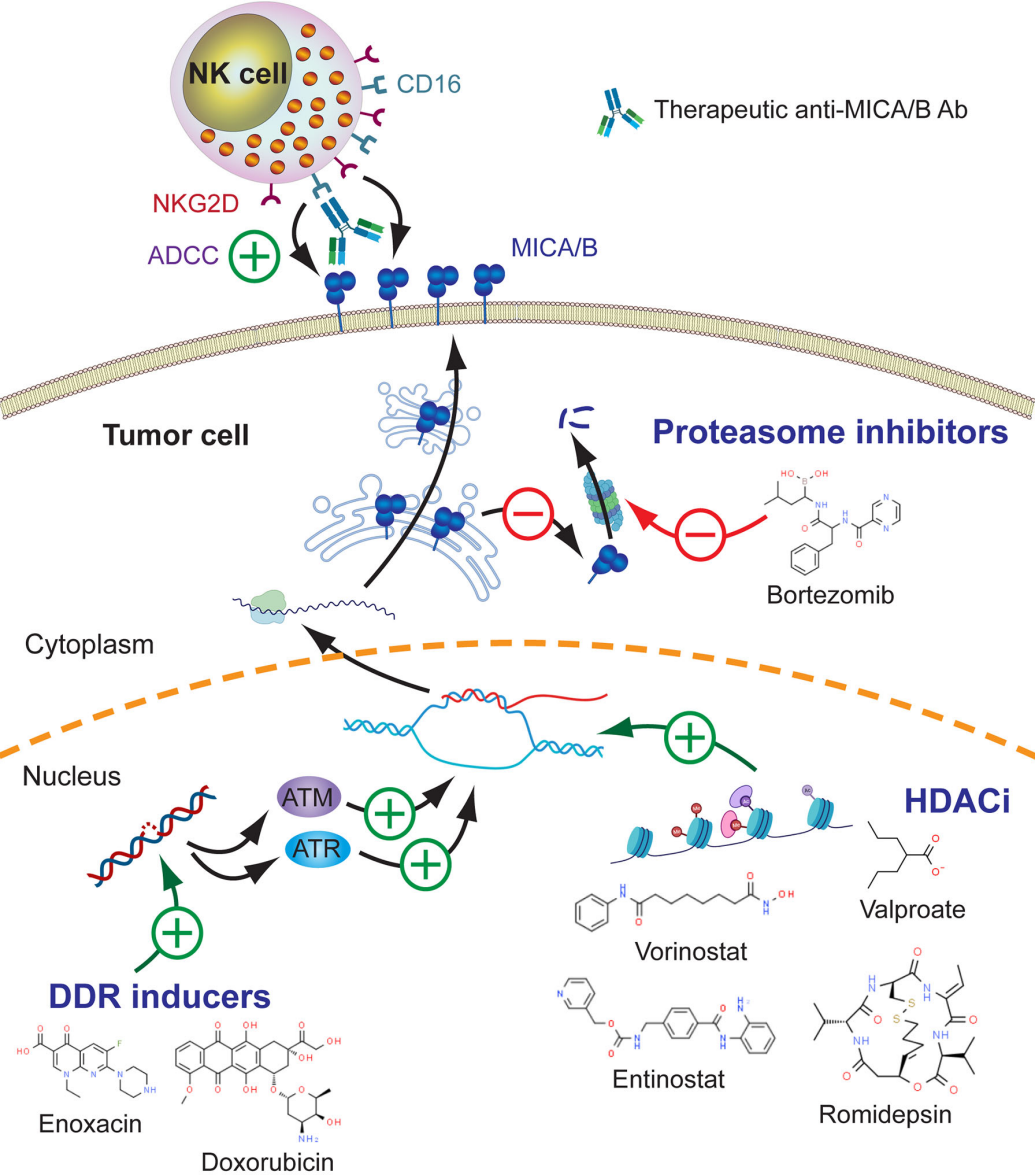
Leveraging anti-MICA/B Ab therapeutic efficacy through combination therapies with DDR inducers, HDACi and proteasome inhibitors. The use of DDR inducers, HDACi and proteasome inhibitors may lead to an increased MICA/B synthesis, reduced degradation, and its consequent accumulation on the cell surface. This effect may result in an improved CD16-dependent ADCC of anti-MICA/B Ab, and a recovery of NKG2D-dependent NK cell-mediated cytotoxicity against tumor cells, facilitating the reinstatement of an efficient tumor cell elimination.

In summary, several drugs currently used to treat patients with different types of cancer might stimulate upregulation of MICA/B which may lead to a subsequent increased sensitivity of tumor cells to the therapeutic effects mediated by anti-MICA/B Ab (ADCC through CD16) and to NK cells through NKG2D. The search for assets that promote upregulated expression of MICA is particularly relevant because tumors evolve under immunological pressure of the host, and such pressure may promote the emergence of tumors with low cell surface expression of MICA/B. Also, MICA/MICB exhibit a very low mutational burden (https://portal.gdc.cancer.gov/), indicating that drug-induced enforced MICA/B expression on tumor cells and targeting with Ab would hardly face the problem of the emergence of escape variants. These effects of DDR inducers, HDACi and proteasome inhibitors on MICA/B expression and their impact on the efficacy of anti-MICA/B Ab are schematically depicted in **Figure 3** and several candidate drugs that are approved, in phase II or late-stage clinical are listed in **Table 1**.

**Table 1:** Approved, phase II and late-stage clinical drugs that might be combined with anti-MICA/B Ab.

Category	Drug/asset	Indication	Status	References	Reported expression of MICA/B^1^
**HDACi**	SAHA (vorinostat)	Advanced NSCLC	FDA approved	(245, 252–255)	+
	PXD101 (belinostat)	Relapsed or refractory PTCL	FDA approved	(245)	NF^2^
	MS-275 (entinostat)	BC (NCT03538171 and NCT02115282)	Late-stage clinical	(253, 255, 267)	+
	LBH589 (panobinostat)	MM	FDA approved	(222)	+
	FR901228 (romidepsin, depsipeptide or FK228)	CTCL and PTCL	FDA approved	(245–247)	NF (CTCL)
					NF (PTCL)
**Proteasome inhibitor**	Bortezomib	MM	FDA approved	(99, 107, 263, 272–275)	+
**Synthetic lethality inducers (PARP1 inhibitors)**	Olaparib	OC, BC, PanC ProC	FDA approved	(281–285)	+ (OC)
					+ (BC)
					+ (PanC)
					+ (ProC)
	Rucaparib	OC, ProC	FDA approved	(281, 282)	+ (OC)
					+ (ProC)
	Niraparib	OC, fallopian tube, and primary peritoneal cancer	FDA approved	(281, 282)	+ (OC)
	Talazoparib	BC	FDA approved	(281, 282)	+
	Veliparib (ABT-888)	BC (NCT02163694, NCT02032277), OC (NCT02470585), Squamous NSCLC (NCT02106546), Non-squamous NSCLC (NCT02264990), GB (NCT02152982)	Late-stage clinical trial	(281, 282)	+ (BC)
					+ (OC)
					+ (NSCLC)
					+ (GB)
**STING agonist**	ADU‐S100/MIW815	HNSCC (NCT03937141)	Phase II	(286—289)	+
**DDR and ICD inducers**	5-fluorouracil	CRC, BC, Gastric Adenocarcinoma, Pancreatic Adenocarcinoma.	FDA approved	(277)	+ (CRC)
					+ (BC)
					+ (GC)
					+ (PanC)
	Doxorubicin	MM, Primary BC, ALL, AML, HL, NHL, Wilms’ tumor, BC, NB, STS, OS, OC, BlC, TC, GC and LC	FDA approved	(272, 290–293)	+ (MM)
					+ (BC)
					+ (ALL)
					+ (AML)
					NF (HL)
					NF (NHL)
					+ (WT)
					+ (NB)
					+ (STS)
					+ (OS)
					+ (OC)
					+ (BlC)
					+ (TC)
					+ (GC)
					+ (LC)
	Melphalan	MM	FDA approved	(272)	+
	Temozolomide (TMZ).	GB	FDA approved	(278)	+
	Epirubicin	BC	FDA approved	(294)	+
	Oxaliplatin	CRC	FDA approved	(294–297)	+
**AXL inhibitor**	Bemcentinib (BGB324)	TNBC and IBC (NCT03184558), LC and NSCLC (NCT03184571), AML and MDS (NCT02488408, NCT03824080), NSCLC (NCT02424617), Mel (NCT02872259), PanC (NCT03649321) , MMeso (NCT03654833)	Phase II	(298–300)	+ (BC)
					+ (LC)
					+ (NSCLC)
					+ (AML)
					+ (MDS)
					+ (Mel)
					+ (PanC)
					+ (MMeso)

^1^Reported expression of MICA/B analyzed by immunohistochemistry and/or flow cytometry in primary tumors.

^2^NF, not found.

ALL, acute lymphoblastic leukemia; AML, acute myeloblastic leukemia; BC, breast cancer; BlC, bladder cancer; CRC, colorectal cancer; CTCL, cutaneous T-cell lymphoma; GB, glioblastoma; GC, gastric cancer; HL, Hodgkin lymphoma; HNSCC, head and neck squamous cell carcinoma; IBC, inflammatory breast cancer; LC, lung cancer; Mel, melanoma; MM, multiple myeloma; MMeso, malignant mesothelioma; NB, neuroblastoma; NHL, non-Hodgkin lymphoma; NSCLC, non-small cell lung carcinoma; OC, ovarian cancer; OS, osteosarcoma; PanC, pancreatic cancer; ProC, prostate cancer; PTCL, peripheral T-cell lymphoma; STS, soft-tissue sarcoma; TC, thyroid carcinoma; TNBC, triple-negative breast cancer.

### Blockers of Shedding of MICA/B

**Figure 4:**
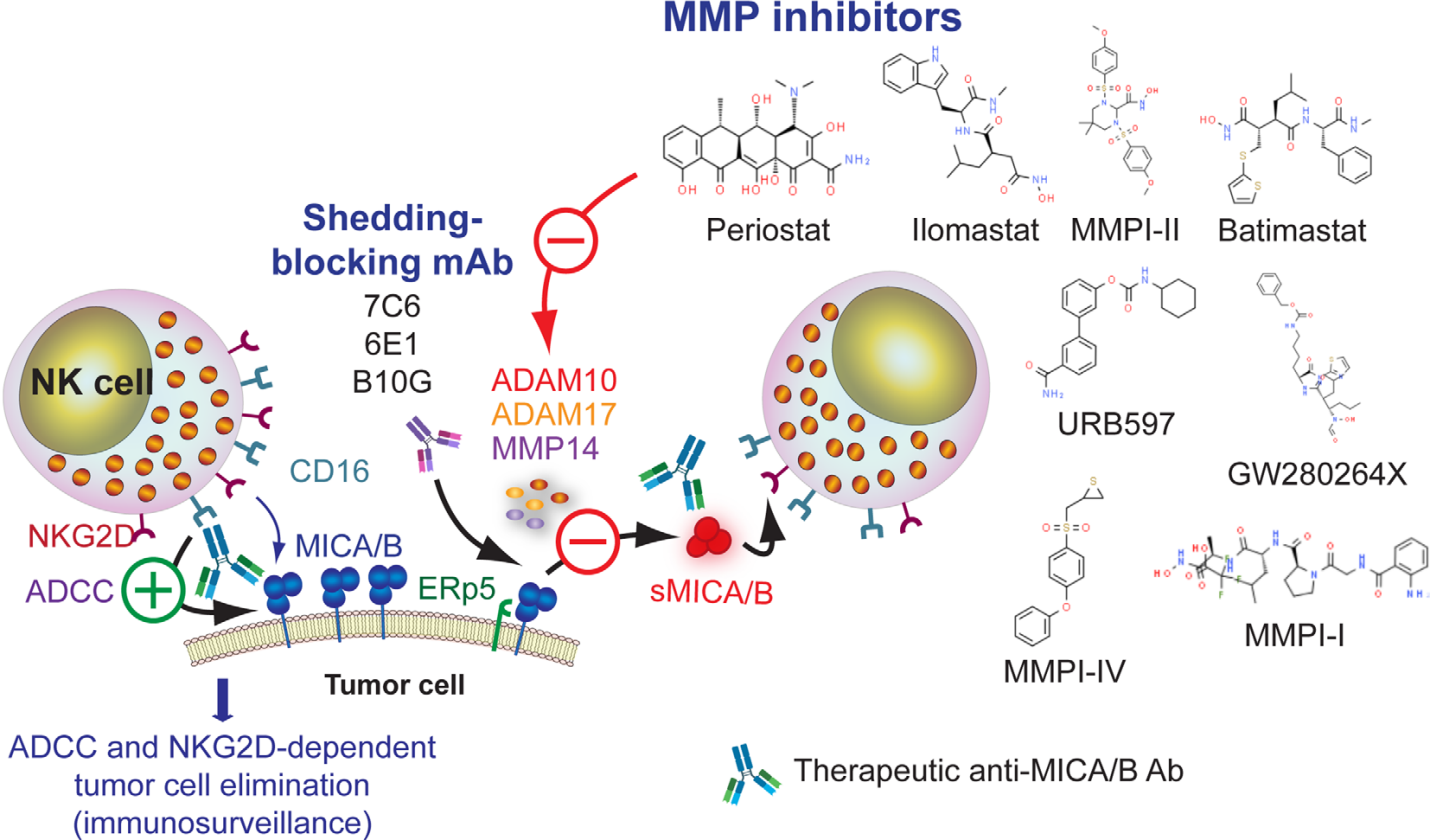
Leveraging anti-MICA/B Ab therapeutic efficacy through combination therapies with pharmacologic inhibition of MMP or with mAb that block MICA/B shedding. Inhibition of MMP with small molecules (detailed in the figure) may prevent MICA/B shedding and lead to a subsequent accumulation of MICA/B on the cell surface. A similar effect can be achieved using mAb that interfere with MICA/B shedding. In both cases, inhibition of MICA/B shedding may lead to an improved CD16-dependent ADCC of anti-MICA/B Ab, and a recovery of NKG2D-dependent NK cell-mediated cytotoxicity against tumor cells.

Shedding of MICA/B depends on proteolytic cleavage mediated by several MMP such as ADAM10, ADAM17 and MMP14 (117, 301, 302) or secretion in exosomes (118, 301). Therefore, blocking the shedding of MICA/B would interfere with the tumor immune escape driven by these soluble forms of MICA/B, resulting also in a restoration of cell surface expression of MICA/B and improved NKG2D-dependent NK cell effector functions (251, 279, 303). Increased cell surface expression of MICA/B would also likely contribute to a better responsiveness to anti-MICA/B Ab and enhanced ADCC. Therefore, MMP inhibitors (MMPI) constitute attractive candidates, which is further supported by the finding that mouse prostate tumors engineered to express a shedding-resistant noncleavable MICB did not grow when implanted into SCID mice but treatment of the animals with an NKG2D blocking mAb led to the development of tumors (304). Several MMPI such as MMPI-I (116, 305), MMPI-II (117), MMPI III (117, 271), MMPI-IV (306), batimastat or BB94 (117, 307), GW280264X (117, 301), GI1254023X (117), ilomastat or GM6001 (117, 301, 308), URB597 (309), periostat or doxycycline (310) and some ADAM-10 selective inhibitors (311, 312) can inhibit MICA/B shedding *in vitro*, resulting in a heightened cell surface expression of the NKG2DL and an increase in NK cell-mediated cytotoxicity. However, among these compounds, only periostat is currently in clinical trials for different types of tumors (313). An alternative approach to inhibit MICA/B shedding is the use of the 7C6 mAb (223) and the 6E1 mAb (119), both of which recognize the α3 domain of MICA/B, inhibit their proteolytic shedding, and lead to increased cell surface expression of MICA/B. Moreover, the B10G5 mAb inhibited the shedding of MICB but its effect on cell surface expression of MICB remains unknown (281, 282). Overall, pharmacologic or mAb-mediated inhibition of MICA/B shedding constitutes another opportunity to combine with anti-MICA/B Ab to induce improved ADCC and tumor cell elimination, as depicted schematically in **Figure 4** and mentioned in **Table 1**.

### Synthetic Lethality Inducers That Target Poly(ADP-Ribose) Polymerase 1

**Figure 5:**
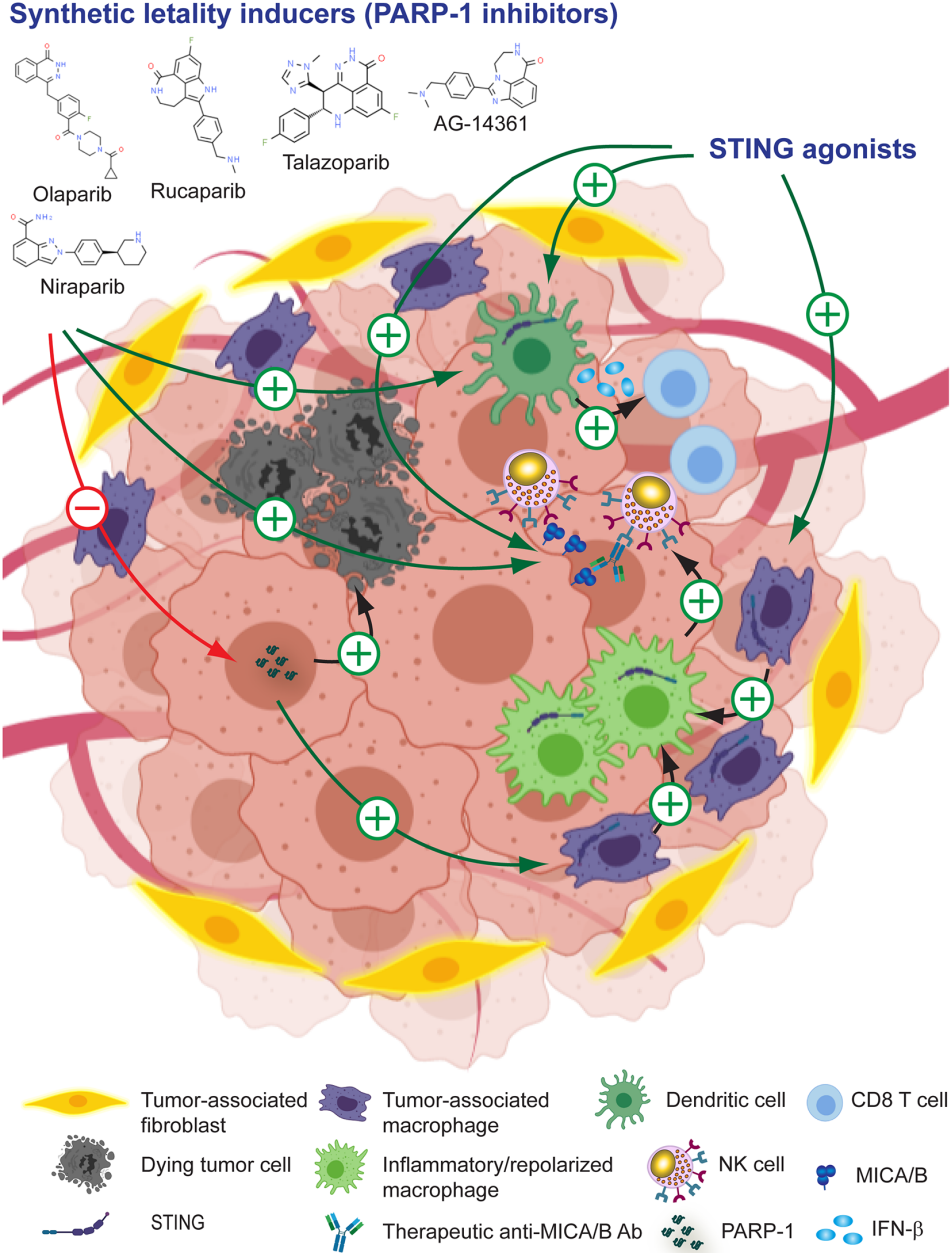
Leveraging anti-MICA/B Ab therapeutic efficacy through combination therapies with pharmacologic PARP1 inhibitors or STING agonists. The use of PARP1 inhibitors can promote tumor cell death and unleash the activation of STING. Immunogenic cell death and STING activation induce the remodeling of the TME, resulting in a heightened production of IFN-β by DC and CTL-mediated tumor eradication, and a reprogramming of TAM into pro-inflammatory macrophages. These pro-inflammatory macrophages, instead of inhibiting NK cells, might now promote efficient NK cell effector functions. In addition, PARP1 inhibition and STING activation might promote increased expression of MICA/B, resulting in an improved CD16-dependent ADCC of anti-MICA/B Ab, and a recovery of NKG2D-dependent NK cell-mediated cytotoxicity against tumor cells. Together, these effects may contribute to foster an efficient tumor cell elimination.

Synthetic lethality inducers such as olaparib, rucaparib, niraparib, talazoparib, veliparib and others exert a therapeutic effect due to their ability to inhibit PARP1, a key enzyme in DNA repair and the preservation of genome integrity (283, 314). However, PARP1 inhibitors also can foster tumor immunity because they activate the cyclic GMP–AMP synthase (cGAS)–stimulator of interferon (IFN) genes (STING) pathway in tumor cells (315, 316). Thus, there are many efforts underway to explore the combination of PARP1 inhibitors with ICI in cancer patients (284, 317–320). PARP1 inhibition has also been demonstrated to affect the NKG2D/NKG2DL axis because PARP1 is involved in the repression of NKG2DL (mainly MICA and MICB) in LSC in patients with AML. Pre-treatment of AML cells with the PARP1 inhibitor AG-14361 resulted in a reduced leukemogenic activity in NSG mice, and administration of AG-14361 to NSG mice xenografted with human AML followed by administration of human NK cells, inhibited leukemogenesis in an NKG2D-dependen manner (133). Therefore, inhibition of PARP1 unleashes NKG2DL expression on AML cells that in turn become more susceptible to NKG2D-dependent, NK cell-mediated effector functions and efficient tumor cell elimination. PARP1 inhibition with olaparib also increases dendritic cell (DC) activation and CTL infiltration in mouse BRCA1-deficient ovarian tumors (315) and in triple negative breast cancer cells (285) through activation of cGAS–STING in tumor cells. Combination of PARP inhibition and CSF-1R blockade enhanced anti-tumor immunity and prolonged survival of BRCA-deficient tumors *in vivo*, indicating that PARP inhibition affects TAM suppressive activity in the TME (321). In addition, combination of cetuximab (anti-EGFR) or avelumab (anti-PD-L1) with olaparib demonstrated that PARP1 inhibition fosters NK cell-mediated ADCC (322). Overall, these results provide a solid rationale for the development of combination therapies between PARP1 inhibitors and Ab that target MICA/B, as depicted schematically in **Figure 5** and several candidate drugs that are approved, in phase II or late-stage clinical are listed in **Table 1**.

### Agonists of STING

Downstream effects of PARP1 inhibition involves the activation of the cGAS-STING pathway (285, 315), and STING enhances NK cell activation and tumor immunity through the production of type I IFN (323–326). Thus, agonists of STING may reconfigure the TME into a proinflammatory milieu and promote the conversion of “cold” tumors into “hot” tumors in part due to an effect on TAM repolarization (327–330). STING activation also cooperates with ICI to foster antitumor immunity (331–333) *via* TAM reprogramming (333). Also, STING agonists can stimulate NK cell-mediated clearance of CD8 T cell-resistant tumors (325) and mobilize tumor-infiltrating myeloid cells to produce IFN-β which in turn activates NK cells (286). In addition, the HDACi entinostat triggers up-regulated expression of MICA/B on tumor cells (255, 257, 269) and activation of STING (334). Therefore, its therapeutic effect could be due to a remodeling of the TME due to a STING-mediated proinflammatory response with an associated repolarization of TAM, and the induction of the expression of MICA/B that would promote improved NKG2D-dependent NK cell effector functions and ADCC mediated by anti-MICA/B Ab. Several available STING agonists such as cyclic dinucleotides (CDN) and their derivates (2´,3´cGAMP, 3´,3´cGAMP, cAIM-derived CDN, cGAMP-derived and others) are currently being tested as monotherapy or in combination with other assets (335). Additional promising STING agonists with antitumor activity have been developed, one of which can be administered orally (287, 336). Also, as the activation of cGAS/STING pathway induces the up-regulation of mouse NKG2DL (289), STING emerges as another attractive molecular target in I-O to leverage NKG2D-dependent NK cell-mediated anti-tumor effects, and to be combined with Ab against MICA/B to manipulate the TME and catalyze tumor immunity, as depicted schematically in **Figure 5** and mentioned in **Table 1**.

### Drugs That Induce Immunogenic Cell Death

**Figure 6:**
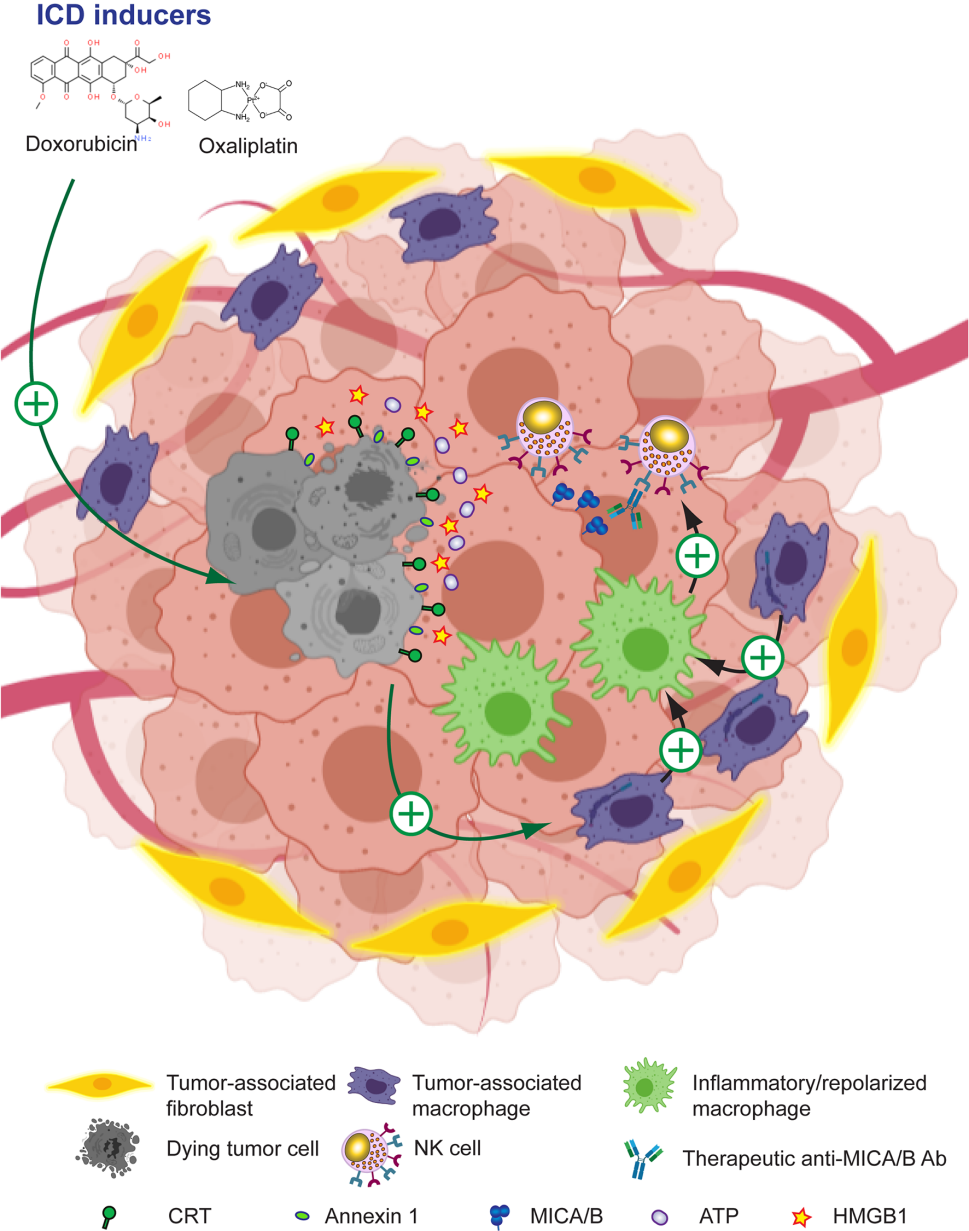
Leveraging anti-MICA/B Ab therapeutic efficacy through combination therapies with ICD inducers. The use of ICD inducers can promote immunogenic tumor cell death with the subsequent expression of CRT and annexin 1 on the tumor cell surface, and the secretion of ATP and alarmins such as HMGB1. Together, these effects result in the remodeling of the TME and a reprogramming of TAM into pro-inflammatory macrophages. These pro-inflammatory macrophages, instead of inhibiting NK cells, might now promote efficient NK cell effector functions such as improved CD16-dependent ADCC of anti-MICA/B Ab, and a recovery of NKG2D-dependent NK cell-mediated cytotoxicity against tumor cells. Together, these effects may contribute to foster an efficient tumor cell elimination.

Drugs that induce ICD such as anthracyclines (doxorubicin, epirubicin, oxaliplatin and others) can trigger an effective antitumor immune response that suppresses tumor growth in mice because they make tumor cells immunogenic. This process involves the mobilization of calreticulin (CRT) to the tumor cell surface, the secretion and extracellular accumulation of ATP and several alarmins such as HMGB1 and annexin A1, and the production of type I IFN which, acting together, contribute to the inflammatory response that remodels the TME, suppress TAM and negatively affect tumor growth (290–293, 295, 337–339). ICD can also be induced by other immunomodulatory compounds such as agonists of RIG-I (340, 341) or STING (288, 342) because they promote the release or expression of several DAMP by tumor cells. ICD treatment of tumors not only turns them intrinsically more immunogenic. Oxaliplatin also promotes the production of T-cell-recruiting chemokines by TAM, resulting in superior CAR-T cell infiltration, remodeling of the TME, and an improved response to ICI (296). Accordingly, many clinical trials currently explore ICD-triggering compounds, alone or combined with ICI, to foster tumor immunity through the stimulation of the immunogenicity of dying tumor cells (294). Moreover, oxaliplatin itself stimulates the upregulation of MICA/B on cancer cells and susceptibility to NK cell-mediated cytotoxicity (297). Therefore, ICD inducers might also be combined with anti-MICA/B Ab to enhance ADCC against MICA/B expressed on tumor cells mediated by NK cells and to prevent their exhaustion through an effect that involves the remodeling of the TME with an associated repolarization of TAM. Moreover, NK cells can themselves promote ICD of tumor cells (343), ICD and NK cell-mediated tumor elimination might be therapeutically amplified to promote a self-perpetuation of the antitumor immune response. For example, CRT exposure on AML blasts is associated with better NK cell-mediated cytotoxicity, and this effect depends on CD11c^+^CD14^high^ cells that become better “helpers” for NK cell activation upon exposure to CRT (337). These concepts indicate that NK cells, through the induction of ICD of tumor cells, can spark-ignite a whole TME remodeling that involves TAM reprograming. Therefore, strategies that potentiate NK ICD and NK cell effector functions such as the use of ICD inducers combined with anti-MICA/B Ab appear as promising alternatives to reinstate tumor immunity, as depicted schematically in **Figure 6** and several candidate drugs that are approved, in phase II or late-stage clinical are listed in **Table 1**.

### Molecules That Target TAM

**Figure 7:**
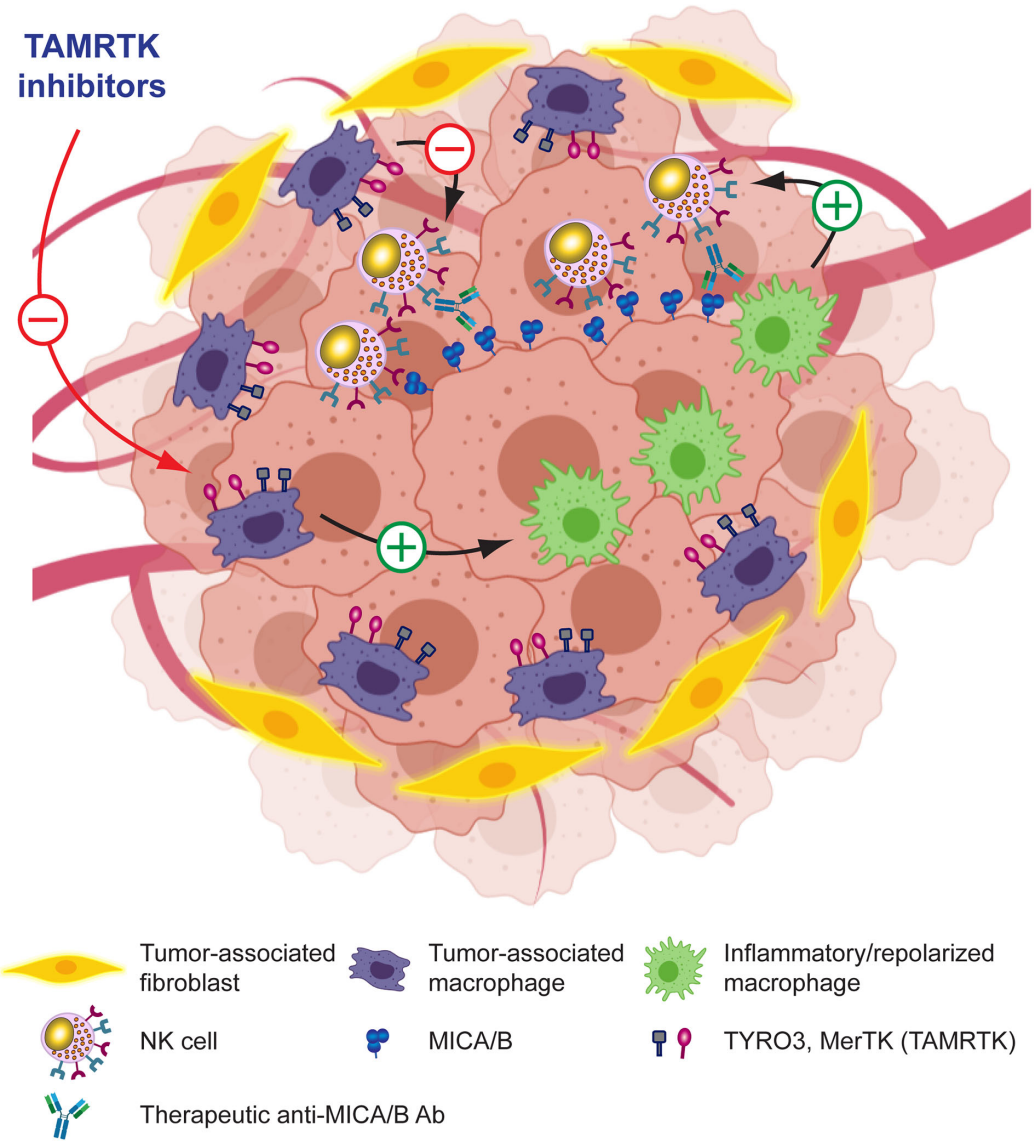
Leveraging anti-MICA/B Ab therapeutic efficacy through combination therapies with molecules that target TAM. Pharmacologic blockade of receptors involved in TAM-mediated immunosuppression in the TME may promote reprogramming of TAM into pro-inflammatory macrophages and a subsequent remodeling of the TME. These pro-inflammatory macrophages, instead of inhibiting NK cells, might now promote efficient NK cell effector functions such as improved CD16-dependent ADCC of anti-MICA/B Ab, and a recovery of NKG2D-dependent NK cell-mediated cytotoxicity against tumor cells. Together, these effects may contribute to foster an efficient tumor cell elimination.

TAM reprogramming into proinflammatory macrophages restores NK cell effector functions *in vitro* and *in vivo* (301–305). To capitalize on TAM reprogramming, several alternatives are being explored. Besides STING agonists and ICD inducers, as discussed earlier, there are additional emerging alternatives. Tyro, Axl and MerTK receptor tyrosine kinases (TAMRTK) constitute important players for the homeostasis of the immune response because they participate in the resolution of inflammation, contribute to the clearance of apoptotic cell debris, the restoration of vascular integrity and regulate the magnitude of the immune response (344). Therefore, interference with TAMRTK function can lead to chronic inflammatory and autoimmunity. Cancer cells coopt regulatory circuits evolutionarily generated to maintain tissue homeostasis in order to resist growth under immunological pressure and promote tumor immune escape. Accordingly, altered expression and signaling of TAMRTK is involved in tumor progression (298, 300) and inhibition of TYRO3 conferred responsiveness to ICI because TYRO3 promotes the development and accumulation of suppressive TAM (345). Also, blockade of MerTK on TAM triggered P2X7R-dependent activation of STING by tumor-derived cGAMP, stimulated a type I IFN response that reshaped the TME, promoted T cell activation and synergized with ICI, contributing to an efficient antitumor immunity (346). Accordingly, Tyro3 or MerTK inhibition interferes with suppressive circuits that are driven by the TME and TAM. Therefore, efforts are being made to develop TAMRTK inhibitors (298–300, 347). Also, it has been demonstrated that TAM exhibit upregulated expression of folate receptor beta (FRβ) within the TME, and that targeting FRβ^+^ TAM with folate coupled to a TLR7 agonist reduced their immunosuppressive activity, stimulated CD8^+^ T-cell infiltration and repolarized macrophages into proinflammatory cells, which negatively impacted on tumor growth and metastasis, and improved overall survival (348). Overall, these results indicate that targeting TAM with TAMRTK inhibitors or other compounds emerge as promising alternatives to reinstate tumor immunity either used as monotherapy or combined with ICI (347). In addition, combination of Tyro3 or MerTK inhibition with anti-MICA/B Ab might synergistically reprogram immunosuppressive TAM, interfere with NK cell exhaustion, and potentiate tumor immunity. Overall, these results provide a solid rationale for the development of combination therapies between pharmacologic reprogramming of TAM and Ab that target MICA/B, as depicted schematically in **Figure 7** and mentioned in **Table 1**.

## Concluding Remarks

NK cells are currently an important component of several pipelines in different pharma/biotech companies aimed at exploiting their therapeutic potential in I-O. Some initial success in the treatment of liquid tumors has been achieved, but solid tumors represent another level of difficulty imposed, among other factors, by the necessity to overcome the suppressive TME in which TAM play a major role. Also, the selection of novel target molecules and therapeutic modalities in I-O is another critical aspect. In this review, we revised data that position MICA and MICB as attractive targets in I-O to leverage NKG2D-dependent NK cell-mediated anti-tumor effects and catalyze tumor immunity. Ab-based strategies emerge as superior to cell-based alternatives because they appear safer, cheaper and of wider application than cell-based therapies such as CAR-T cells or CAR-NK cells. However, as MICA and MICB are polymorphic, therapeutic Ab should target monomorphic/conserved regions. Cell surface MICA/B can be targeted with Ab, either monoclonal or polyclonal, to promote tumor cell elimination through ADCC. Moreover, their soluble counterparts involved in tumor immune escape can be targeted with Ab to promote clearance of immune complexes by macrophages and reinstate tumor immunity. Thus, Ab-based strategies constitute “two-in-one” therapeutic options that might be further fostered through combination with other assets, in a field where combination strategies are taking the center of the stage. Current evidence indicates that such anti-MICA/B Ab can be either monoclonal or polyclonal (for example, induced by immunization with immunogens such as BLS-MICA). Along this review, we presented frontrunner alternatives to combine with these anti-MICA/B Ab such as the use of drugs that can promote upregulation of MICA/B on tumor cells, blockers of sMICA/B shedding, synthetic lethality inducers that target PARP1, agonists of STING, drugs that induce ICD and molecules that target TAM. Directly or indirectly, all these strategies leverage MICA/B in I-O. Overall, most of these strategies may contribute, in a direct or in an indirect manner, to tumor elimination through restoration of NK cell activation and effector functions, and to subsequently convert “cold” tumors into “hot” tumors. Also, some of the discussed strategies such as the use of drugs that trigger upregulation of MICA/B, that promote a remodeling of the TME and/or that affect TAM may also foster CAR-T/CAR-NK cell-based therapies aimed at targeting MICA/B. A central question that remains unanswered is whether it is better to reinvigorate dysfunctional TINK or to eliminate/deplete them and create a niche for the recruitment of newly activated, fully functional NK cells through the administration of immunotherapeutic agents to the patient. In any case, pursuing the addressed pipelines might lead to innovative modalities of immunotherapy for the treatment of a wide range of cancer patients. Moreover, although we focused the state of the art and perspectives on the control of primary tumors and considering that NK cells also play an important role in the detection and eradication of tumor cells within the circulation and limiting metastasis (349–351), the approaches proposed may also impact on the ability of NK cells to suppress metastasis.

## Author Contributions

NZ conceived and designed the review and wrote the manuscript. MF and CD provided critical inputs and ideas and corrected the manuscript. All authors contributed to the article and approved the submitted version.

## Funding

This work was funded with grants from the National Agency for Promotion of Science and Technology from Argentina (ANPCYT), the National Research Council of Argentina (CONICET) and the Trust in Science Program from GlaxoSmithKline (GSK), all to NZ. The funders were not involved in the study design, collection, analysis, interpretation of data, the writing of this article or the decision to submit it for publication. We also thank to Fundación Williams and Fundación René Barón for providing financial assistance (donations) to our laboratory.

## Conflict of Interest

The authors declare that the research was conducted in the absence of any commercial or financial relationships that could be construed as a potential conflict of interest.

## Publisher’s Note

All claims expressed in this article are solely those of the authors and do not necessarily represent those of their affiliated organizations, or those of the publisher, the editors and the reviewers. Any product that may be evaluated in this article, or claim that may be made by its manufacturer, is not guaranteed or endorsed by the publisher.
